# A Deep Learning Model for Predicting Patient Age and Disease Status Using Pattern ERG Data

**DOI:** 10.1093/geroni/igaf122.3688

**Published:** 2025-12-31

**Authors:** Hunter Porter, Jonathan Wren

**Affiliations:** Oklahoma Medical Research Foundation, Oklahoma City, Oklahoma, United States; Oklahoma Medical Research Foundation, Oklahoma City, Oklahoma, United States

## Abstract

The eye is an extension of the central nervous system and can act as a window into neural, vascular, and metabolic health. One measure of retinal function, the pattern electroretinogram (PERG), is used to assess central vision by presenting patients a patterned stimulus (e.g. – an alternating checkerboard). These data have proven useful in understanding a variety of conditions such as diabetic retinopathy, glaucoma, and age-related macular degeneration. However, PERG data currently requires expertise in both performing and interpreting the assay. We hypothesized that an artificially intelligent (AI) model could help with PERG interpretation and potentially provide novel insights outside of ocular dysfunction. We used a publicly available dataset of human PERG traces (PERG-IOBA) to train a deep-learning AutoEncoder (AE) model by tasking it with reproducing real sample data. Then, we used the rich encoder portion of the AE as input for new models tasked with predicting patient age and clinician-annotated disease status which were evaluated on left out test data. These predictor models showed great accuracy in identifying normal from abnormal PERG traces (AUC = 0.834) and were much better age predictors (mean absolute error = 11.7 years) than the naïve model (MAE = 14.8 years). Our next steps involve collecting data from a variety of clinicians to ensure generalizability and to predict non-ocular conditions (e.g. – Alzheimer’s and diabetes status). Finally, we want to share these models to enable earlier identification of functional deficits and broader adoption of formerly specialist-only assays such as PERG.

